# Normal Visual Acuity and Electrophysiological Contrast Gain in Adults with High-Functioning Autism Spectrum Disorder

**DOI:** 10.3389/fnhum.2015.00460

**Published:** 2015-08-26

**Authors:** Ludger Tebartz van Elst, Michael Bach, Julia Blessing, Andreas Riedel, Emanuel Bubl

**Affiliations:** ^1^Section for Experimental Neuropsychiatry, Department for Psychiatry and Psychotherapy, Albert-Ludwigs-University, Freiburg, Germany; ^2^Section Visual Function, Eye Center, University Medical Center, Freiburg, Germany

**Keywords:** autism spectrum disorder, visual acuity, contrast gain, Asperger’s syndrome, vision

## Abstract

A common neurodevelopmental disorder, autism spectrum disorder (ASD), is defined by specific patterns in social perception, social competence, communication, highly circumscribed interests, and a strong subjective need for behavioral routines. Furthermore, distinctive features of visual perception, such as markedly reduced eye contact and a tendency to focus more on small, visual items than on holistic perception, have long been recognized as typical ASD characteristics. Recent debate in the scientific community discusses whether the physiology of low-level visual perception might explain such higher visual abnormalities. While reports of this enhanced, “eagle-like” visual acuity contained methodological errors and could not be substantiated, several authors have reported alterations in even earlier stages of visual processing, such as contrast perception and motion perception at the occipital cortex level. Therefore, in this project, we have investigated the electrophysiology of very early visual processing by analyzing the pattern electroretinogram-based contrast gain, the background noise amplitude, and the psychophysical visual acuities of participants with high-functioning ASD and controls with equal education. Based on earlier findings, we hypothesized that alterations in early vision would be present in ASD participants. This study included 33 individuals with ASD (11 female) and 33 control individuals (12 female). The groups were matched in terms of age, gender, and education level. We found no evidence of altered electrophysiological retinal contrast processing or psychophysical measured visual acuities. There appears to be no evidence for abnormalities in retinal visual processing in ASD patients, at least with respect to contrast detection.

## Introduction

Autism spectrum disorder (ASD) is a common variant of a neurodevelopmental disorder with an estimated prevalence above 1% (Levy et al., 2009). According to ICD-10 and DSM-5 definitions, specific patterns in social perception, social competence, and communication skills, as well as a strong need for routines and highly circumscribed interests, are the core features of ASD (Dakin and Frith, 2005)^1^. However, there is increasing awareness that perceptual features are also central to ASD (Jemel et al., 2010; Markram and Markram, 2010). Individuals with ASD frequently report perceptual hypersensitivity, such as perceiving loud noises or bright light as aversive. In the visual domain, a preference for focusing on small items and details is well recognized (Gillberg, 2003; Rogers and Ozonoff, 2005).

Given these observations, there has been discussion regarding whether alterations in the neurophysiology of basic perceptions might be more critical in the pathophysiology of ASD than previously thought. Following this line of reasoning, a number of papers have focused on possible alterations in the basic visual capacities of ASD patients (Gillberg, 2003; Dakin and Frith, 2005; Rogers and Ozonoff, 2005; Simmons et al., 2009).

As early as 1940s, Kanner (1943) and Asperger (1944) described perceptual symptoms as the core features of autism. Now, approximately 70 years later, these symptoms are, indeed, included in the DSM-5 diagnostic criteria (American Psychiatric Association, 2013; Tebartz van Elst, 2013). Nonetheless, the precise nature and role of these symptoms in the pathophysiology of autism remains unclear.

Although reports of an “eagle-eye” kind of supersensitive visual acuity in ASD have attracted tremendous attention, such reports have since been questioned on the basis of methodological flaws (Ashwin et al., 2009; Bach and Dakin, 2009). In another line of visual research alterations, at the level of the occipital cortex in visual evoked potentials (VEPs) to Gabor contrast stimuli (Pei et al., 2014), isolated bright and dark checks (Weinger et al., 2014) as well as event-related responses to small and large checker-board stimuli were recently reported (Kornmeier et al., 2014). However, hitherto, most of these reports have not yet been replicated in large samples.

In a series of experiments, we analyzed various early visual signals of different neuropsychiatric conditions to explore the possible objective markers of these conditions. In ADHD, a neurodevelopmental disorder with close associations with ASD, we found alterations in the electrophysiological measures of retinal background noise (Bubl et al., 2015a). In depression, a frequent comorbidity of adult ASD, we found the electrophysiological retinal contrast gain to be significantly reduced (Bubl et al., 2015b), as measured by the pattern electroretinogram (PERG) (Bubl et al., 2010) and VEP (Bubl et al., 2015b). These signal abnormalities normalized following successful therapy (Bubl et al., 2012).

Against the background of these findings, the aim of the present study was twofold: (1) we wanted to further the exploration of possible alterations in early visual information processing in ASD (i.e., visual acuity and a hyperacuity, namely, Vernier acuity); (2) we wanted to test the specificity of findings concerning early visual processing in ADHD and depression by measuring the same signals in ASD; and (3) we wanted to evaluate both visual contrast gain and visual background noise by employing the PERG.

## Materials and Methods

### Subjects

Following the approval of the ethics committee of the Albert-Ludwigs-Universität Freiburg, all patients were recruited from the Freiburg Center for Diagnosis and Treatment of Autism (University Center for Autism Spectrum, Universitäres Zentrum Autismus Spektrum Freiburg, UZAS; http://www.uniklinik-freiburg.de/psych/live/patientenversorgung/schwerpunkte/schwerpunkt-asperger.html). For the present study, we included only those patients fulfilling the diagnostic criteria for Asperger’s syndrome (AS), according to ICD-10 (ICD-10 F84.5) and DSM-IV (299.80). The diagnostic process was organized according to the recommendations of the NICE Guidelines for Adult Autism (National Institute for Health and Clinical Excellence: Autism in Adults; full guidelines in http://guidance.nice.org.uk/CG142/NICEGuidance/pdf/English). Specifically, the clinical diagnoses of ASD and AS were established through the consensus diagnosis of a multi-professional team following a structured diagnostic procedure. The clinical diagnosis was based on a thorough, generally multi-session history exploration of each patient, which focused on the patient’s development of autistic symptoms throughout his or her life. A history of caregivers (e.g., parents, partners, siblings, etc.) and behavioral observations was also an essential component of this process, which usually took several sessions of two or more hours. Psychometric tools included the following instruments, which were put into routine use prior to clinical assessment: the autism spectrum quotient (AQ) (Baron-Cohen et al., 2001), the empathy quotient (EQ) (Baron-Cohen and Wheelwright, 2004), the Bermond–Vorst alexithymia questionnaire (BVAQ) (Vorst and Bermond, 2001), the WURS (Retz-Junginger et al., 2002), Conners’ Adult ADHD Rating Scales Self-Report: Long version (CAARS-S:L), and the Becks depression inventory (BDI) (Hautzinger et al., 1994); in addition, instruments, such as autism diagnostic interview-revised (ADI-R) (Lord et al., 1994) and the autism diagnostic observation schedule-generic (ADOS) (Lord et al., 2000). Alternatively, behavioral assessments were employed in selected and unclear in-patient cases. The same is true of additional neuropsychological tests assessing executive and theory-of-mind capacities. The multi-professional diagnostic team consisted of three experienced senior consultant psychiatrists and two fully qualified senior psychologists. The final diagnosis was made through a consensus of all persons involved in the diagnostic process, which invariably included at least two experienced consultant psychiatrists or psychologists. Study control participants were also assessed clinically, and they completed the AQ and EQ questionnaires. The mean years of schooling was assessed for all participants to determine the general level of intelligence. Patients with relevant medical or neurological diseases aside from depression and anxiety, as well as those with histories of schizophrenia, ADHD, bipolar disorder, or any other psychiatric Axis I disorder, were excluded from the study. Patients with ASD and current depressive episodes or anxiety disorders were also excluded.

### Visual acuities: Optotype acuity and hyperacuity assessment

Visual acuity (optotype based) and visual hyperacuity (Vernier acuity) were assessed psychophysically using the Freiburg vision test (FrACT) (Bach, 2007b). This test has been employed in hundreds of studies. Standard visual acuity, here denoted as “optotype acuity,” was tested with a Landolt C through an adaptive staircase procedure. The results were converted to the standard unit “logMAR” (the logarithm of the minimum angle of resolution), which is normally distributed (in contrast to the Snellen fraction) – but which, unfortunately, runs counter intuitively, such that more negative values correspond to better acuity. Acuity charts are typically spaced in 0.1 logMAR steps from line to line. For hyperacuity, we assessed the Vernier acuity, finding the existence of two vertical lines that were nearly collinear but that had a slight horizontal offset. This offset is known as Vernier acuity (Walls, 1943; Levi et al., 1985), and it is 5–10 times higher than the standard optotype acuity. The Vernier lines had no vertical gap (“abutting condition”), and the horizontal offset was judged by the participant, namely, whether the top line was to the left or right of the bottom line. To avoid movement cues, the line pair was offset horizontally for each of the 42 presentations per run. The threshold Vernier offset (75% correct) was determined by an adaptive staircase procedure, and the results were obtained as log(arcseconds) of the threshold offset.

Freiburg vision test has a 95% limit-of-agreement range of ±0.2 logMAR (Bach, 2007b). For the Vernier test, we found a 95% limit-of-agreement test-retest range of ±0.33 logArcsec.

The acuity stimuli were presented on a special high-resolution organic light-emitting diode-display (OLED-display) at 57 cm distance.

All subjects had a visual acuity greater than 20/25 (=lower than +0.1 logMAR), with appropriate correction at the distance used for visual stimulation (Bach, 2007b).

### Stimulation and electrophysiological measurement

For stimulation, recording, and analysis, we used the EP2000 system (Bach, 2000). The stimuli were generated with a resolution of 800 × 600 pixels at a frame rate of 75 Hz in a dimly lit room. They were displayed on a monitor covering a field size of 32° × 27.0° at an observation distance of 57 cm, with a mean stimulus luminance of 45 cd/m^2^. The patients were refracted as necessary for the observation distance. To ensure appropriate fixation and accommodation, the patients reported digits that appeared at random intervals in place of the fixation target.

To evoke the PERG, a sequence of six contrast-reversing (at 12.5 reversals per second) checker-board stimuli with check sizes of 0.51°, were presented with Michelson contrasts of 1, 3.2, 7.3, 16.2, 36, and 80%. Each contrast level was presented for 10 s before the next contrast was applied; finally, the test returned to the first contrast level. This interleaved sequence was presented until 80 artifact-free trials per contrast (each 0.96 s in length, containing 12 responses) were accumulated. The interleaved blocking ensured that any sequential effects (e.g., fatigue) were distributed equally across all contrast values. This protocol was repeated once, and further analysis was based on the vector average of each pair of recordings.

DTL electrodes (Bach, 2007a; see also www.michaelbach.de/dtl.html), which were placed at the lower limbus of each eye, were used to record the PERG simultaneously from both eyes. These were referenced to gold cup electrodes at the ipsilateral outer canthi, with one earlobe grounded. Subjects were asked to blink infrequently during the recording and to maintain a relaxed pose. Sweeps exceeding ±130 μV were rejected as artifacts, and the number of artifacts per condition was saved with the PERG data.

The signals were amplified, filtered (first order 0.5–100 Hz), and digitized at 1 kHz with 16-bit resolution. To prevent temporal aliasing, all timings (e.g., stimulation, analog sampling, and sweep length) were related to the stimulus monitor frame rate (Bach et al., 1997; Bach and Meigen, 1999). The duration of the recording was approximately 1 h per subject.

### Analysis of the electrophysiological data

Offline, all traces were first de-trended (such that the trend remaining was mainly from blink excursions) through the calculation of a linear regression along the trace, which was subtracted to avoid the possibility of sawtooth artifacts mimicking background noise (Bach et al., 1997; Bach and Meigen, 1999). Then, the magnitude spectrum was calculated through a discrete Fourier transform. Based on the analysis interval of 0.96 s, the spectrum starts at 1.04 Hz, is spaced at 1.04 Hz intervals, and contains responses with a specific reversal rate (12.5 Hz), harmonics (25, 37.5 Hz, etc.) and background noises (non-stimulus-driven neural activity) at all frequencies. There is occasional mains interference at 50 Hz. From this spectrum, a noise-free response estimate was calculated (Bach and Meigen, 1999; Meigen and Bach, 1999), and the background noise was taken as the average of the two spectral magnitudes next to the target signal at 12.5 Hz (at 11.46 and 13.54 Hz). A linear regression of the spectral response magnitude of the target signal at 12.5 Hz versus the stimulus contrast, forced through zero contrast and amplitude, yielded the PERG-based contrast gain, as defined by the slope of the fit (Bubl et al., 2010). The PERG amplitude versus the contrast is essentially linear (Hess and Baker, 1984; Thompson and Drasdo, 1994; Bach and Hoffman, 2006; Bach et al., 2013), and this slope is termed the “PERG contrast gain” or the “contrast gain” throughout the paper. The non-linear superposition of noise and response magnitude was first analyzed by Strasburger (1987) in the context of steady-state VEP recording, which also applies to steady-state PERG recording. When strict integer relations for all pertinent frequencies are selected (see note on temporal aliasing above), this spectrum contains response power only at the stimulus frequency and its harmonics. Norcia et al. (1989) used a single spectral line, offset from their response frequency by 2 Hz. Since the average of the two adjacent frequencies above and below the response frequency is an even better estimator of background noise (Meigen and Bach, 1999), this method was employed here. The overall background noise ratio was established using the average noise level across all contrast levels and both eyes (Bubl et al., 2015a).

Possible differences in visual acuities, the PERG contrast gain, and the retinal background noises of the two groups were calculated using MANCOVA. The influences of age and medication on our signals were analyzed as covariates in the MANCOVA. Pearson’s rank correlations were used to assess the relationships among visual acuity, PERG contrast gain, PERG-based background noise, and the psychometric properties. A *p*-value of 0.05 was chosen as the criterion of significance.

## Results

### Group comparison

The present study included 33 individuals with high-functioning ASD, as well as 33 group-matched control subjects. In the ASD group, 23 subjects took no psychotropic medication, and the remaining 10 took antidepressant medication (i.e., SSRI, SNRI, or SNRI with Mirtazapin). None of the subjects in the control group were taking any psychotropic medication.

There were significant differences in psychometric scores between the two groups (Table 1). The Vernier acuity was assessed in 22 ASD and 28 control subjects.

**Table 1:** **Summary of demographic and psychometric data**.

	ASD (*n* = 33)	Controls (*n* = 33)	Statistics
	Mean [SEM]	Mean [SEM]	
Age	39.5 [1.9]	34.4 [2.1]	*T* = 1.8; *p* = 0.076
Gender	11:25	12:21	χ^2^ = 0.273; df = 1; *p* = 0.34
Mean years of schooling	12.5 [0.2]	12.2 [0.2]	*T* = 1.1; *p* = 0.29
AQ	38.0 [1.1]	13.9 [0.9]	*T* = 16.1; *p* < 0.001
EQ	16.4 [1.7]	43.0 [1.9]	*T* = −11.0; *p* < 0.001
BDI	12.5 [1.7]	2.7 [0.5]	*T* = 5.6; *p* < 0.001
WURS	45.7 [2.6]	20.8 [2.2]	*T* = 7.5; *p* < 0.001
CAARS-SL	16.8 [1.6]	9.1 [0.9]	*T* = 4.3; *p* < 0.001
Previous depression	24		
No comorbidity	9		

### Demographic and psychometric data

Table 1 summarizes the demographic and psychometric data for all participants. There was no significant difference among the groups in terms of age, gender, or years of schooling. However, the mean age of the control group was lower than that of the ASD group (*p* = 0.076), which was why we controlled for age in all subsequent statistical group comparisons.

### Main outcome measures

A comparison of the two groups revealed no significant difference for our main outcome measures after correcting for age and medication (Table 2).

**Table 2:** **Summary of main outcome measures**.

	ASD patient	Control	MANCOVA
	Mean [SEM]	Mean [SEM]	
Visual acuity OD [logMAR]	−0.06 [0.04]	−0.09 [0.02]	*F* = 1.884; df = 1; *p* = 0.18
Visual acuity OS [logMAR]	−0.10 [0.02]	−0.10 [0.02]	*F* = 1.131; df = 1; *p* = 0.29
Vernier acuity [log(arcsec)]	1.09 [0.07]	1.17 [0.05]	*F* = 3.369; df = 1 *p* = 0.073
PERG contrast gain [μV/100%]	2.92 [0.18]	3.21 [0.16]	*F* = 0.276; df = 1; *p* = 0.60
Background noise	0.085 [0.01]	0.082 [0.01]	*F* = 0.346; df = 1; *p* = 0.56

*OD, oculo dexter (right eye); OS, oculo sinister (left eye)*.

Quaid et al. (2002) reported a correlation coefficient between Vernier acuity and optotype acuity of *r* = 0.8 for normal subjects. In our total population, we find that *r* = 0.52 (*p* < 0.001); for our controls, we find *r* = 0.55 (*p* = 0.0025); and for ASD, we find *r* = 0.57 (*p* = 0.0057). Thus, roughly half of the variance was common to these two types of acuities; this left room for differential effects between ASD and control, which we did not observe.

**Figure 1:**
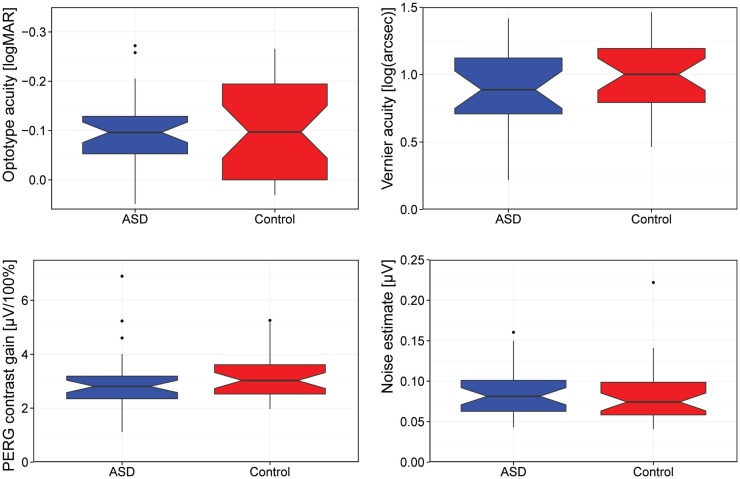
**Comparison of visual acuity (logMAR), Vernier acuity [log(arcseconds)], contrast gain, and background noise**. [Box-plot details: the median is indicated by the thick horizontal lines, the notches represent a 95% confidence interval for the medians, the box covers the 25–75% percentile range, the “antennas” indicate the range, and the outliers (1.5 times the interquartile distance beyond the quartiles) are indicated by circles.]

Figure 1 summarizes our main results as a box-plot illustration.

### Medication effect on outcome measures

When comparing the effects of medication on our outcome measures, as before, we did not find significant differences in the covariate medication in the MANCOVA [visual acuity OD (*F* = 2.799; *p* = 0.099), visual acuity OS (*F* = 2.036; *p* = 0.159), Vernier hyperacuity (*F* = 1.965, *p* = 0.168), contrast gain (*F* = 0.084; *p* = 0.772), and background noise (*F* = 0.686, *p* = 0.411)].

### Effect of age on the outcome measures

In the MANCOVA, the covariate age had a significant effect on the contrast gain [*F* = 8.024, *p* = 0.006; visual acuity OD (*F* = 5.840, *p* = 0.019)] in both groups. Age had no effect on visual acuity OS (*F* = 0.774, *p* = 0.382), Vernier acuity (*F* = 2.141, *p* = 0.150), or noise (*F* = 0.244, *p* = 0.623) in our sample.

### Dimensional relationship between outcome measures and ASD symptom scores

**Figure 2:**
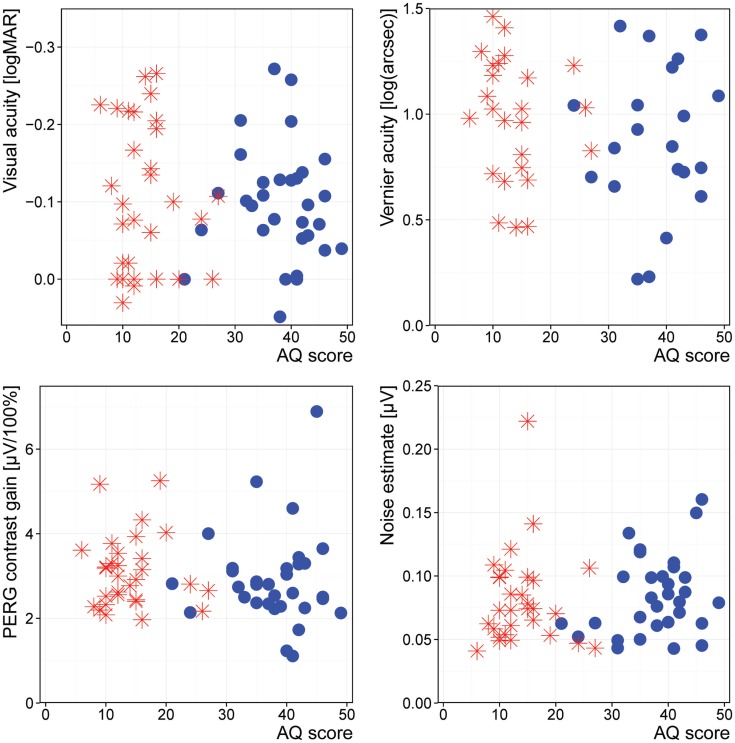
**Association of AQ with our outcome measures**. Stars: controls; disks: ASD.

The correlation between our target measures and the measures for the severity of ASD revealed no significant differences (Figure 2).

## Discussion

In this study, we compared different measures of psychophysical and electrophysiological early visual information processing, which ranged from visual acuity to retinal contrast perception, between individuals with ASD and control subjects. Neither the psychophysiological visual acuity nor the Vernier hyperacuity differed between the ASD participants and the control subjects. Furthermore, there were no changes in the electrophysiological processing of retinal contrast stimuli with respect to retinal contrast gain or retinal background noise.

The retina represents a network of very early visual information processing, with strong modulatory effects from different neurotransmitters. Our negative findings illustrate that the function of the retina is not compromised – at least not in the subgroup of high-functioning subjects with ASD – without evidence for comorbid neuropsychiatric disorders (Tebartz van Elst et al., 2013).

### Relationship with other findings

In this study, we analyzed a battery of markers of early visual information processing. In comparing our findings with those of previous research in the literature, the focus must be directed separately to the different signals of interest.

#### Electrophysiological Findings

##### Retinal contrast gain

To our knowledge, there have been no prior studies investigating PERG signals in patients with ASD. Therefore, our study must be regarded as a pilot study in this respect. In contrast to our findings for persons with depression (Bubl et al., 2010) and with ADHD (Bubl et al., 2013), we did not find signal abnormalities. Consequently, there seems to be some specificity to the finding of reduced PERG contrast gains in depression, since we did not detect such abnormalities in ADHD (Bubl et al., 2013) or ASD in this study.

Nonetheless, these findings are not replicated. Furthermore, one has to consider that contrast gain may depend on precise stimulus characteristics. For example, in children, a specific signal abnormality of contrast stimuli between 5 and 17 cpd has been reported, suggesting the special relevance of stimulus resolution. Therefore, the missing difference between the ASD and the control groups might be due to suboptimal stimulation resolutions. Moreover, our data are not comparable to those of Ritvo et al. (1988), who found reduced flash-ERG b-wave amplitudes in 13 of 27 autistic persons. However, we studied individuals with very high-functioning ASD in relation to a diagnosis of AS, while Ritvo et al. (1988) analyzed individuals with autistic disorder and much lower IQs. Furthermore, the flash ERG does not assess the same retinal physiology as the PERG. While the former predominantly represents the activity of rod and cone cells [i.e., a- and b-waves (Preiser et al., 2013)], the latter predominantly reflects the physiology of the retinal ganglion cells (Maffei and Fiorentini, 1981; Bach and Hoffman, 2006; Bach et al., 2013).

##### Retinal background noise

In a recent study on ADHD, we found evidence for an elevated signal-to-noise ratio for the PERG signal in this neurodevelopmental condition, which is also very common in ASD (Hofvander et al., 2009). In addition, a VEP study on children with ASD reported an elevated signal-to-noise ratio and background noise at the level of the occipital cortex (Weinger et al., 2014).

By contrast, in our sample, we did not elicit any change in retinal background noise. Therefore, based on our data (Bubl et al., 2013), there seems to be some specificity to this signal, since it was abnormal for ADHD, but not for ASD. In this context, it is noteworthy that our ASD patients did not suffer from comorbid ADHD, since we excluded this comorbidity in the clinical interview. Nevertheless, some ASD participants scored higher in the CAARS-SL, which we believe results from the fact that the questions in the CAARS also address problems experienced by participants with ASD, resulting in elevated, false positive CAARS-SL scores.

#### Psychophysiological Findings

##### Visual acuity

Recently, there has been discussion concerning the possibility of “eagle-eyed-vision” in ASD, based on a report (Ashwin et al., 2009) whose methodology was found to be flawed (Bach and Dakin, 2009; Crewther and Sutherland, 2009). In our study, we did not find a better-than-normal visual acuity in ASD. This finding is consistent with other recent relevant reports (Milne et al., 2009; Tavassoli et al., 2011; Bölte et al., 2012; Albrecht et al., 2014). The one exception (Brosnan et al., 2012) again used flawed methodology (Bach, personal communication). One report found superior mean Vernier acuity in ASD (Latham et al., 2013), but only for the “separated,” non-abutting condition, which was not tested here. Vernier hyperacuity is a 5–10 times finer measure of visual acuity, and it is closely connected to the neural mechanism in the visual cortex (Fahle et al., 1995). In our study, although we did not find superior Vernier acuity in ASD, we did find slightly (albeit non-significantly) reduced values. The missing difference between the two groups indicates that the preference for small details does not derive from perceptual alterations in this domain (Koldewyn et al., 2013).

Our findings regarding different signals of early vision are further supported by the lack of association between any psychometric scores and our outcome measures. None of the signals of early vision – visual acuity, hyperacuity, retinal contrast gain, or retinal background noise – were correlated with the psychometric measures of autistic symptoms.

##### Medication effect

Medication taken by the patients did not predict our outcome measures. This suggests that the medication taken by the patients did not modulate visual acuity, retinal contrast gain, or background noise.

### Methodological issues

This study has limitations that need to be considered. The patient group presented with a higher mean BDI, which is fairly typical for adults with high-functioning ASD (Hofvander et al., 2009; Riedel et al., 2015). However, none of the patients suffered from a clinical diagnosis of acute depression.

Some authors suggest that the stimulus distance of the Vernier acuity measurement has special relevance in ASD patients; however, this distance was not varied in this study (Latham et al., 2013). The lines in the Vernier acuity test can either be closely attached or present with a gap between the lines. Latham et al. (2013) found no difference in the abutting Vernier paradigm, though they reported a significant change when the Vernier lines were separated. While the Vernier difference between the ASD group and the controls in this study falls just short of significance, we cannot exclude the possibility that a change in the Vernier paradigm possibly might have resulted in a significant difference between the two groups.

In a previous study (Pei et al., 2014), electrophysiological changes were found at spatial frequencies between 5 and 17 cpd, which are higher than the stimuli used here (our check size of 0.51° corresponds to spatial frequencies of roughly 1.38 cpd). Therefore, differences in early visual processing for ASD might appear at other spatial frequencies.

### Possible implications of our findings

We conclude that the clinical observation of a preference for small visual objects is, at a minimum, not linked to those signals that were measured in this study. Visual acuity is widely determined by the optical system of the eye, and especially the density of the photoreceptors. While Vernier hyperacuity is linked to the cortical function, it is still an early perceptual quality (Fahle et al., 1995). Neither was compromised in our study.

Therefore, our observations support the assumption that changes in visual perception in ASD are organized in higher cortical areas (Kornmeier et al., 2014; Weinger et al., 2014), a notion that might be supported by reports of VEP alterations in response to small stimuli in such patients (Kornmeier et al., 2014; Pei et al., 2014). Further systematic research will help to pinpoint the precise level of alterations of visual information processing in ASD. For that purpose, studies, such as ours, which analyze the earliest steps of such neurophysiological processes, provide critical pieces of evidence.

### Summary

In summary, this study found no evidence of altered PERG contrast gain, background noise, or attenuated visual acuity. At least with respect to contrast detection, there is no evidence for abnormalities in retinal visual processing in persons with ASD with normal intelligence. The ability to perceive distinct details in ASD must be organized in higher visual circuits.

## Author Contributions

LT, MB, JB, AR, and EB contributed to the article in the conception, design, analysis, and interpretation of data, drafting the article, and revising it critically for important intellectual content. In giving final approval of the version to be published, they are accountable for all aspects of the work in ensuring that questions related to the accuracy or integrity of any part of the work are appropriately investigated and resolved.

## Conflict of Interest Statement

The authors declare that the research was conducted in the absence of any commercial or financial relationships that could be construed as a potential conflict of interest.

## Footnotes

^1^Since many different studies from the Freiburg research groups are published using similar visual assessment methods and patient samples with ASD, this paper may overlap in wording and phrasing with other papers produced by our group using the same sample methods or ASD patient samples, particularly with regard to the description of methodological procedures.
